# Supplementary material for: Taccalonolide AJ uncouples tubulin GTP hydrolysis from microtubule depolymerization
Source: bioRxiv. 2026 Oct 1:2026.09.30.755716. Preprint. [Version 1] doi: 10.64898/2026.09.30.755716 (PMC13648680; doi:10.64898/2026.09.30.755716)
Supplement: Supplementary file 1 [file media-1.pdf]

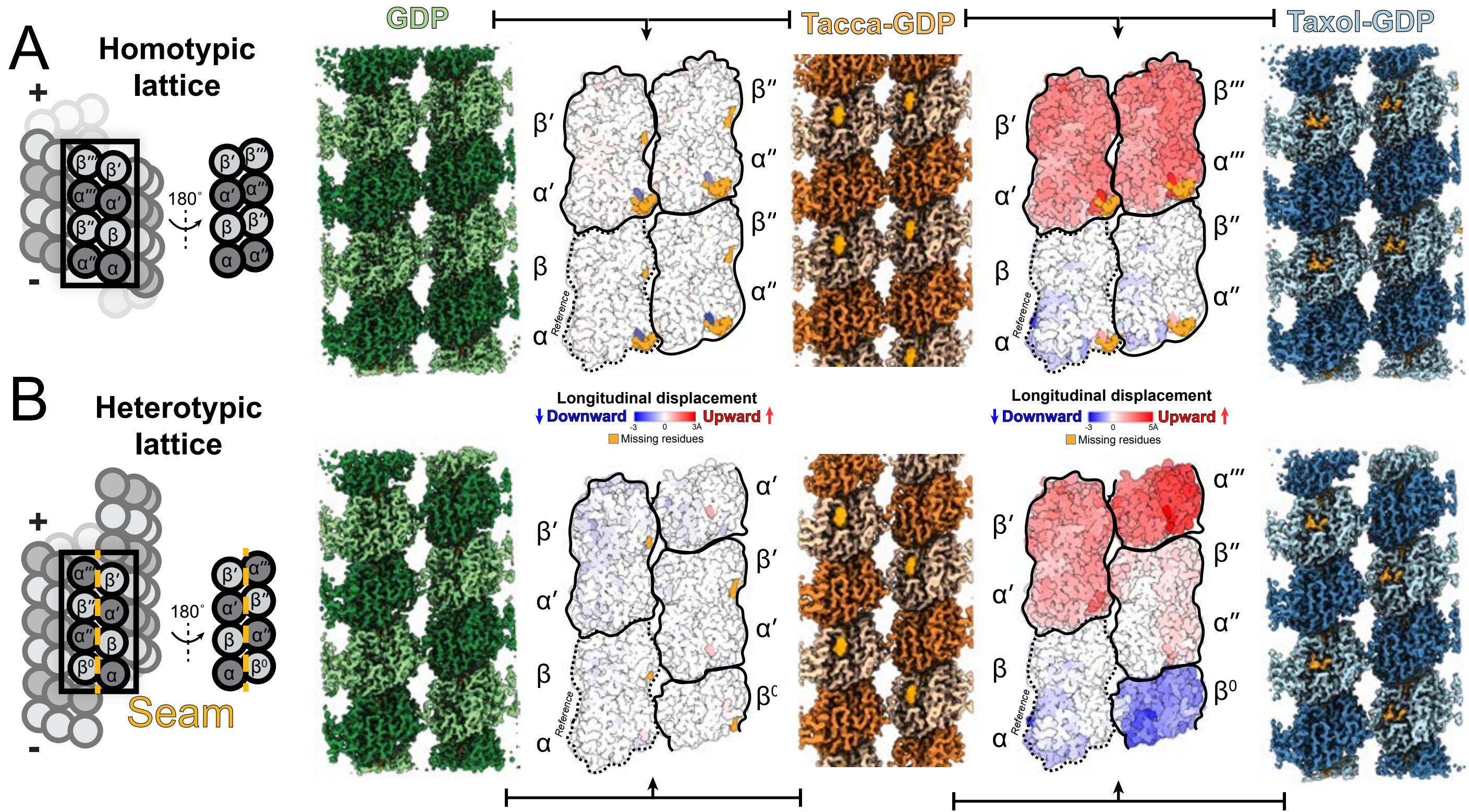

Extended Data Figure 1 - Comparison of longitudinal conformational changes between taccaAJ-GDP, GDP, and paclitaxel-GDP.

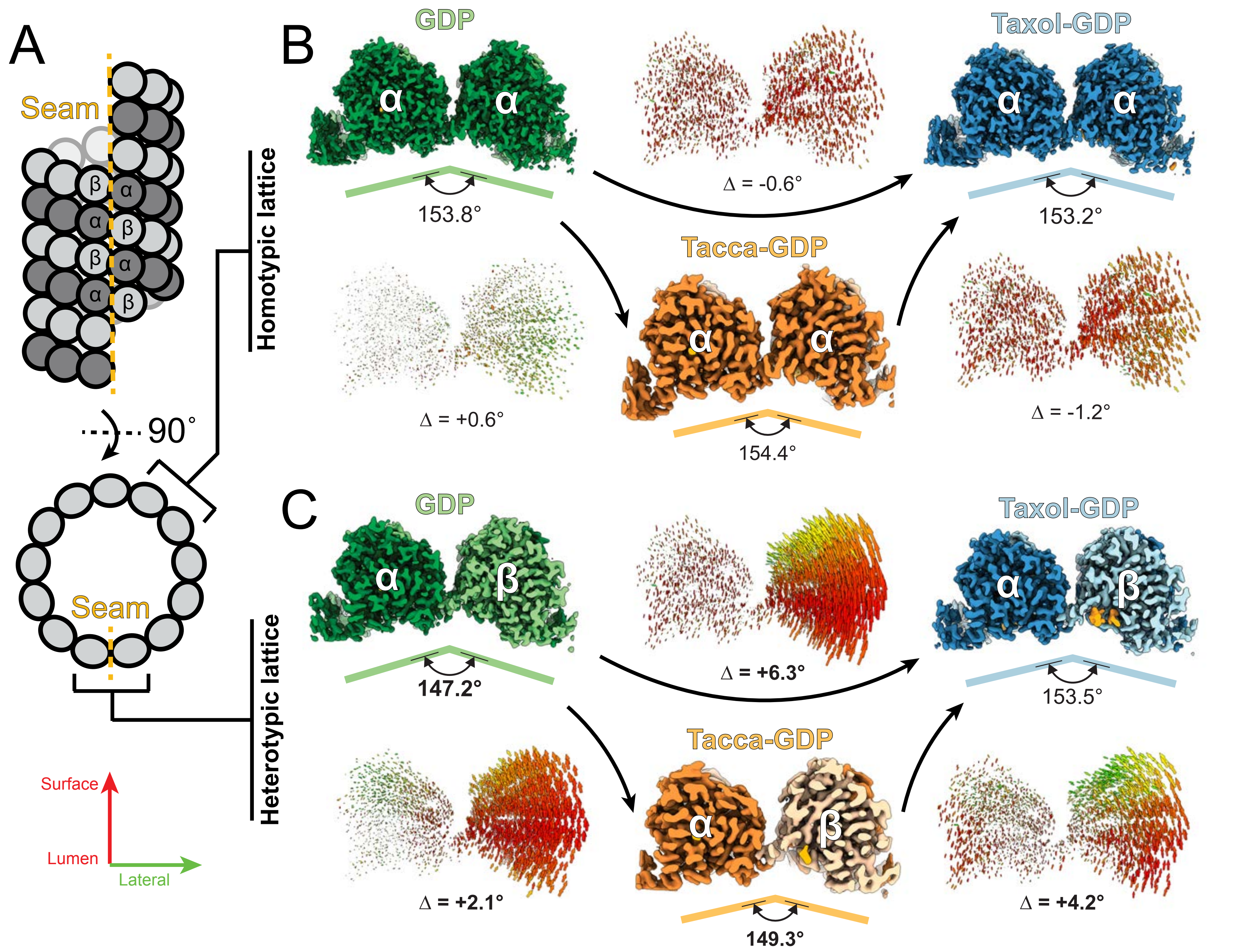

Extended Data Figure 2 - Comparison of lateral changes between taccaAJ, GDP, and paclitaxel-GDP.

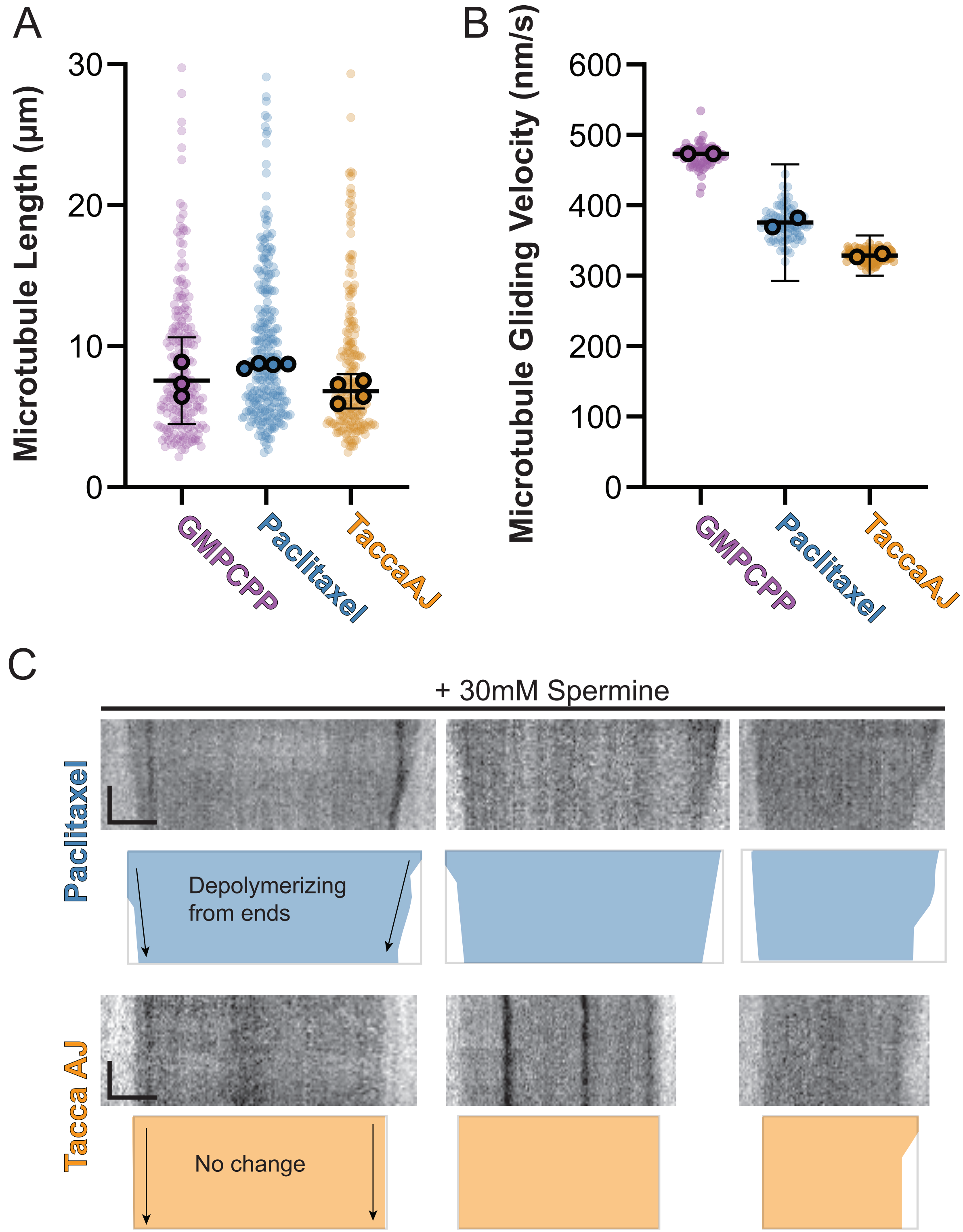

Extended Data Figure 3 - Gliding assay and spermine treatment of microtubules.

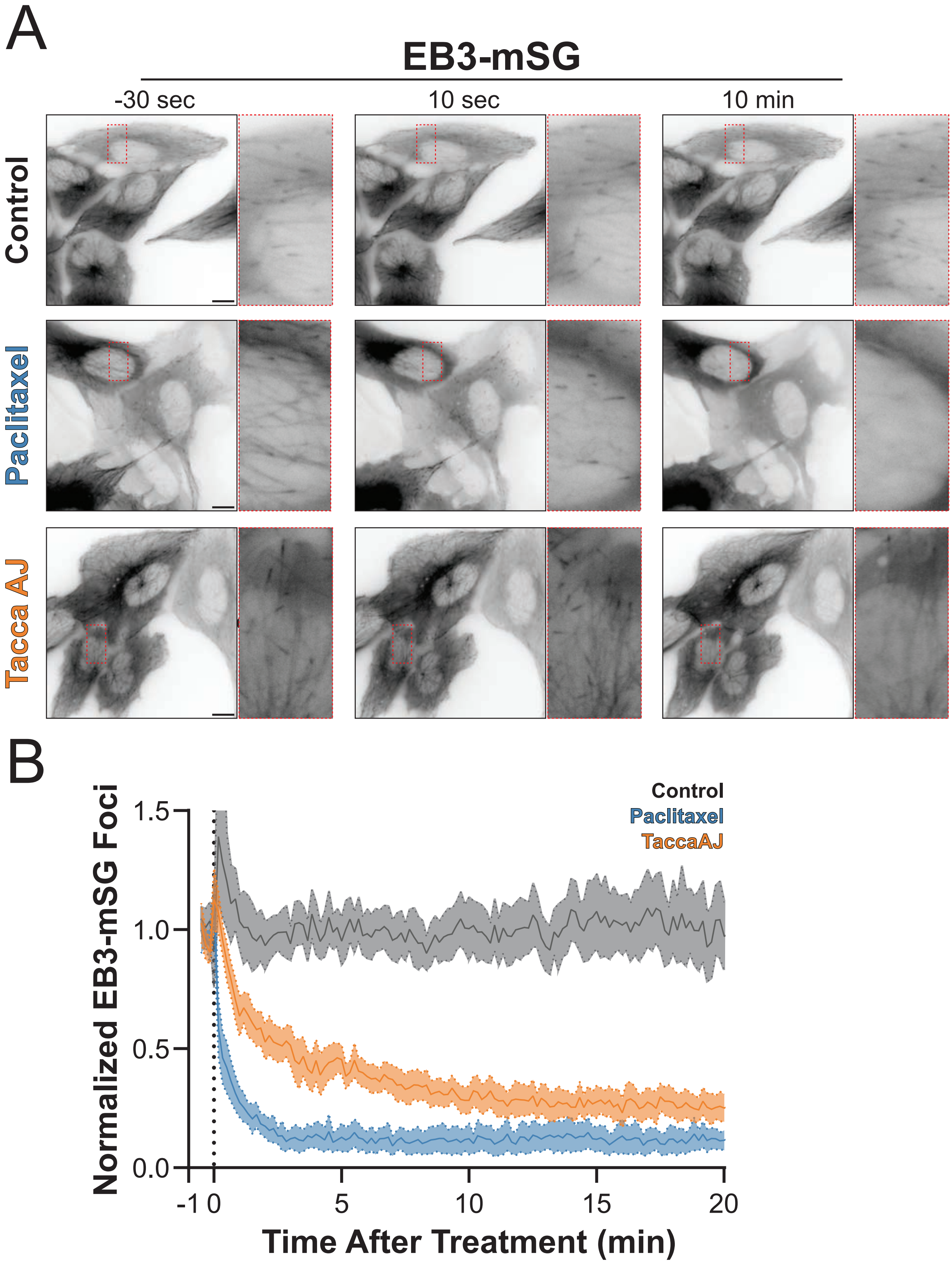

Extended Data Figure 4 - TaccaAJ quickly disrupts MT dynamics in cells

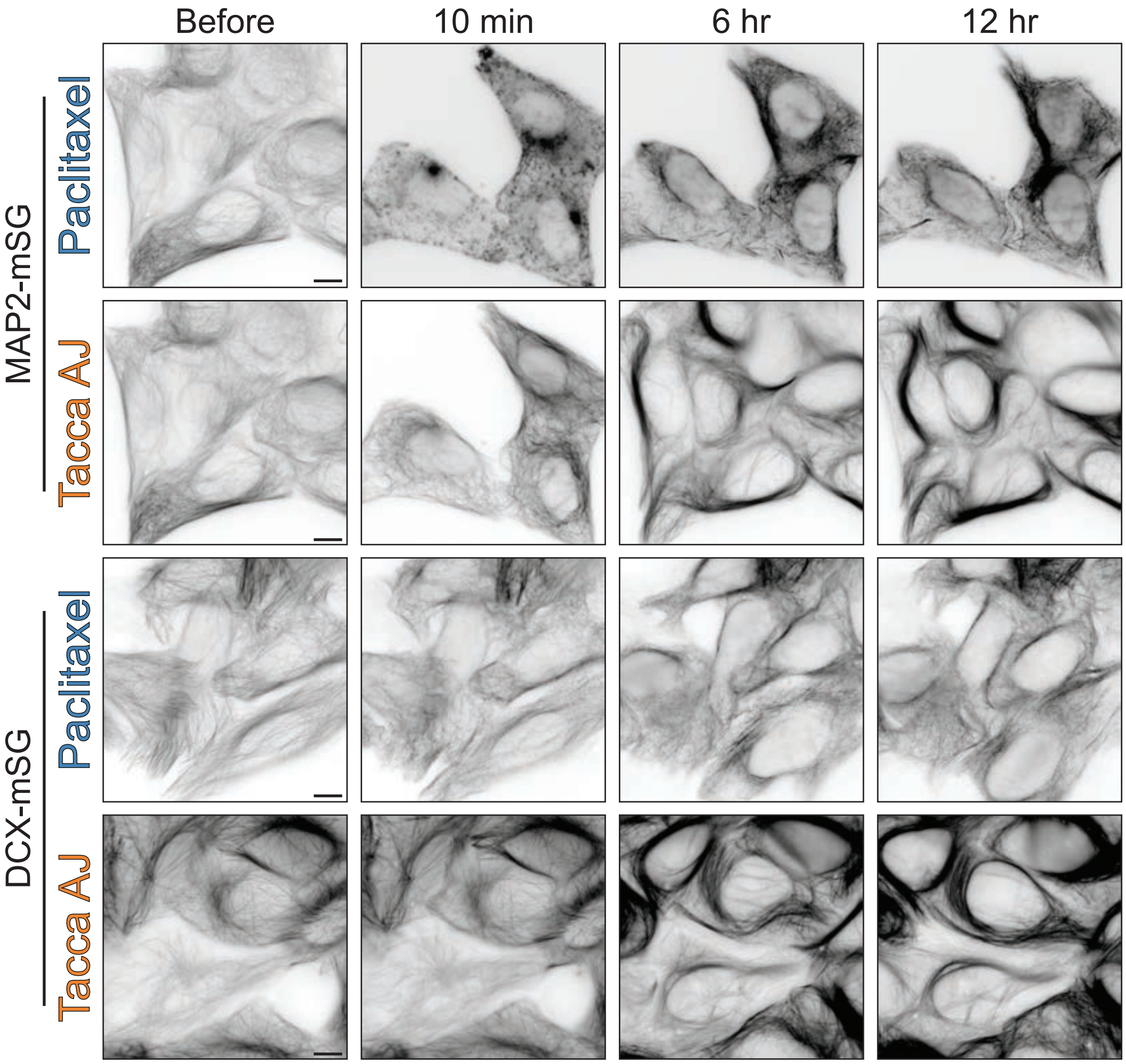

Extended Data Figure 5 -Tacca AJ-stabilized MTs remain compacted for 12 hours

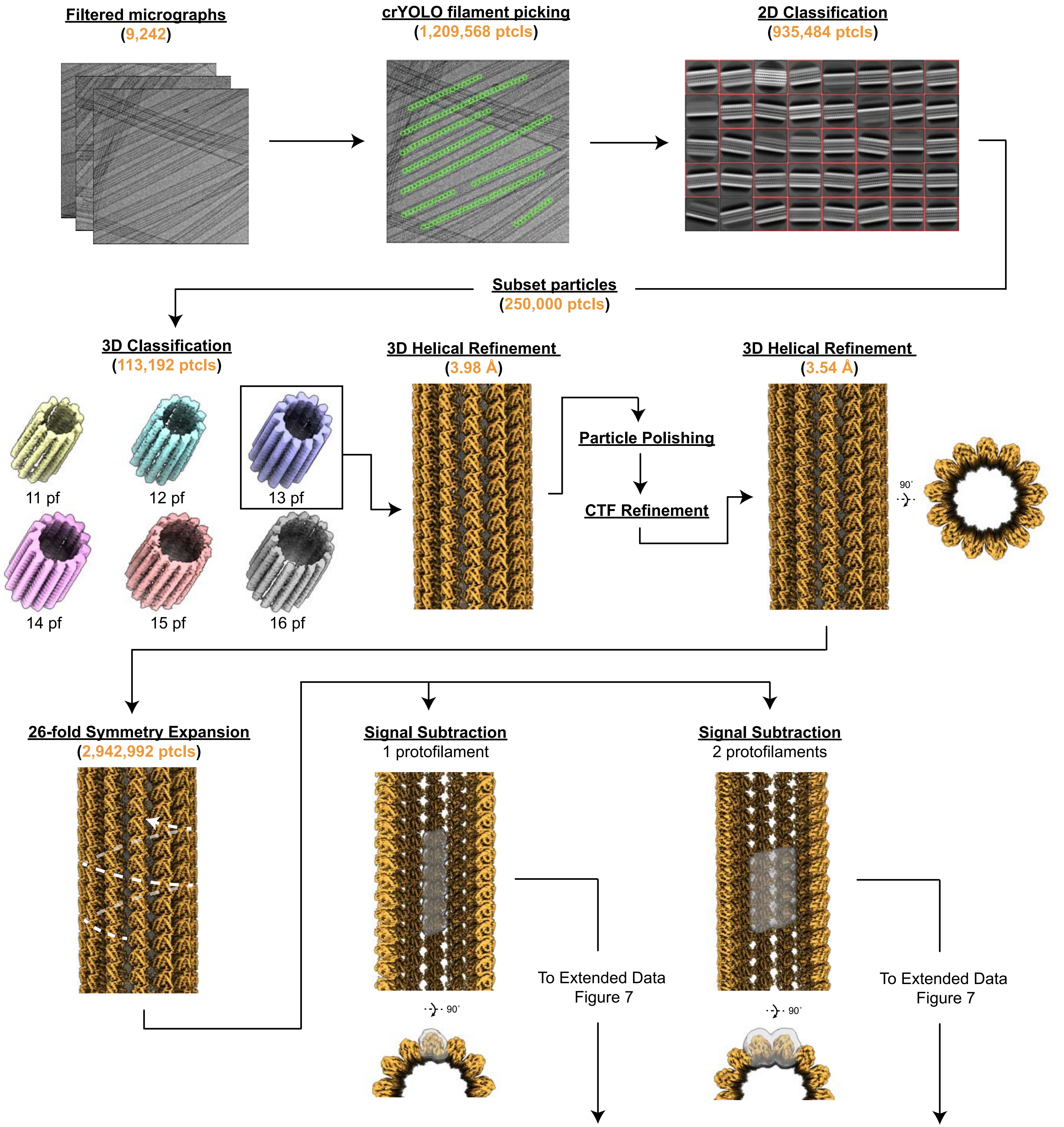

Extended Data Figure 6 - TaccaAJ-GDP whole-microtubule cryo-EM data processing workflow.

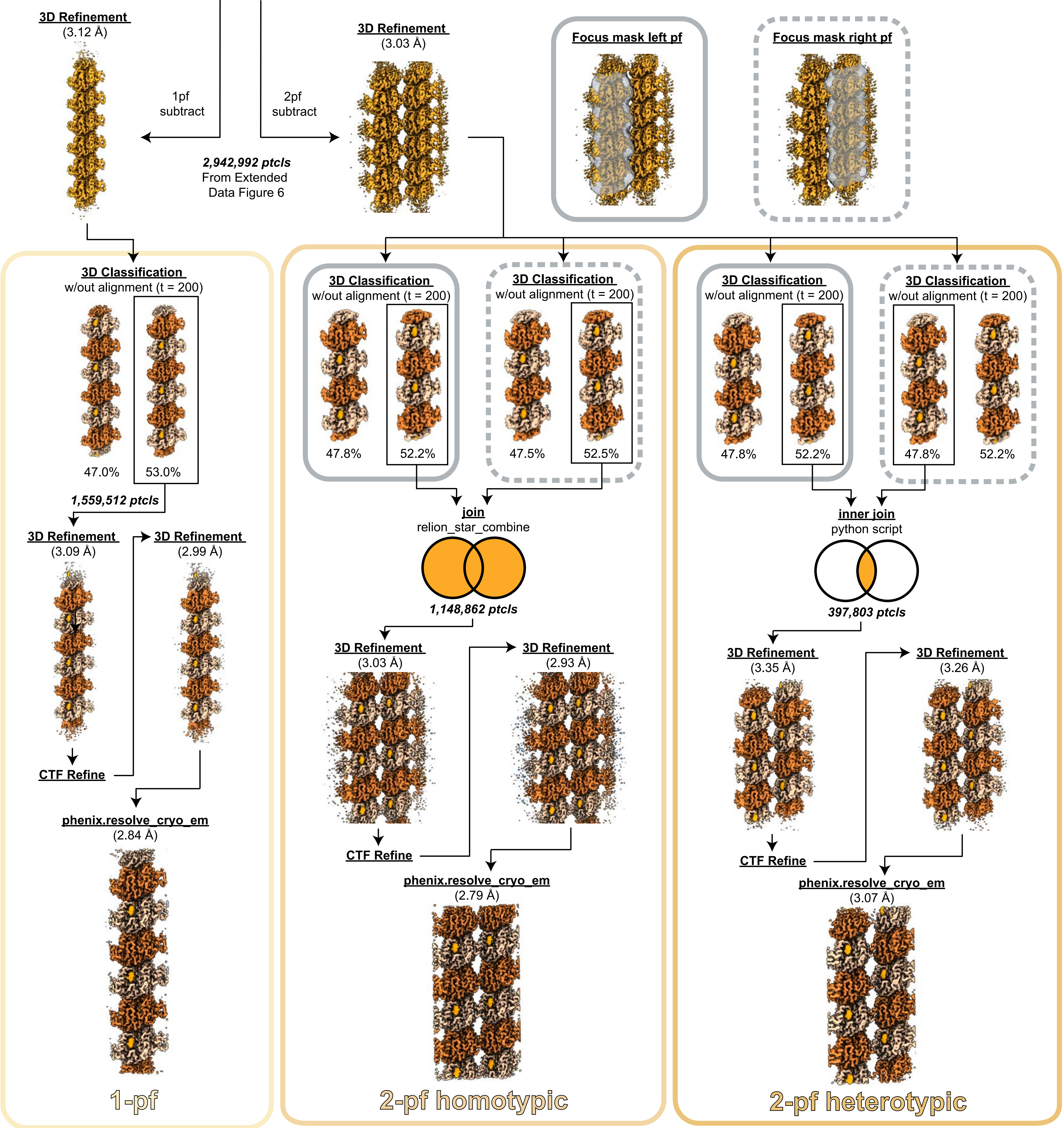

Extended Data Figure 7 - TaccaAJ-GDP 13-protofilament microtubule cryo-EM data processing workflow.

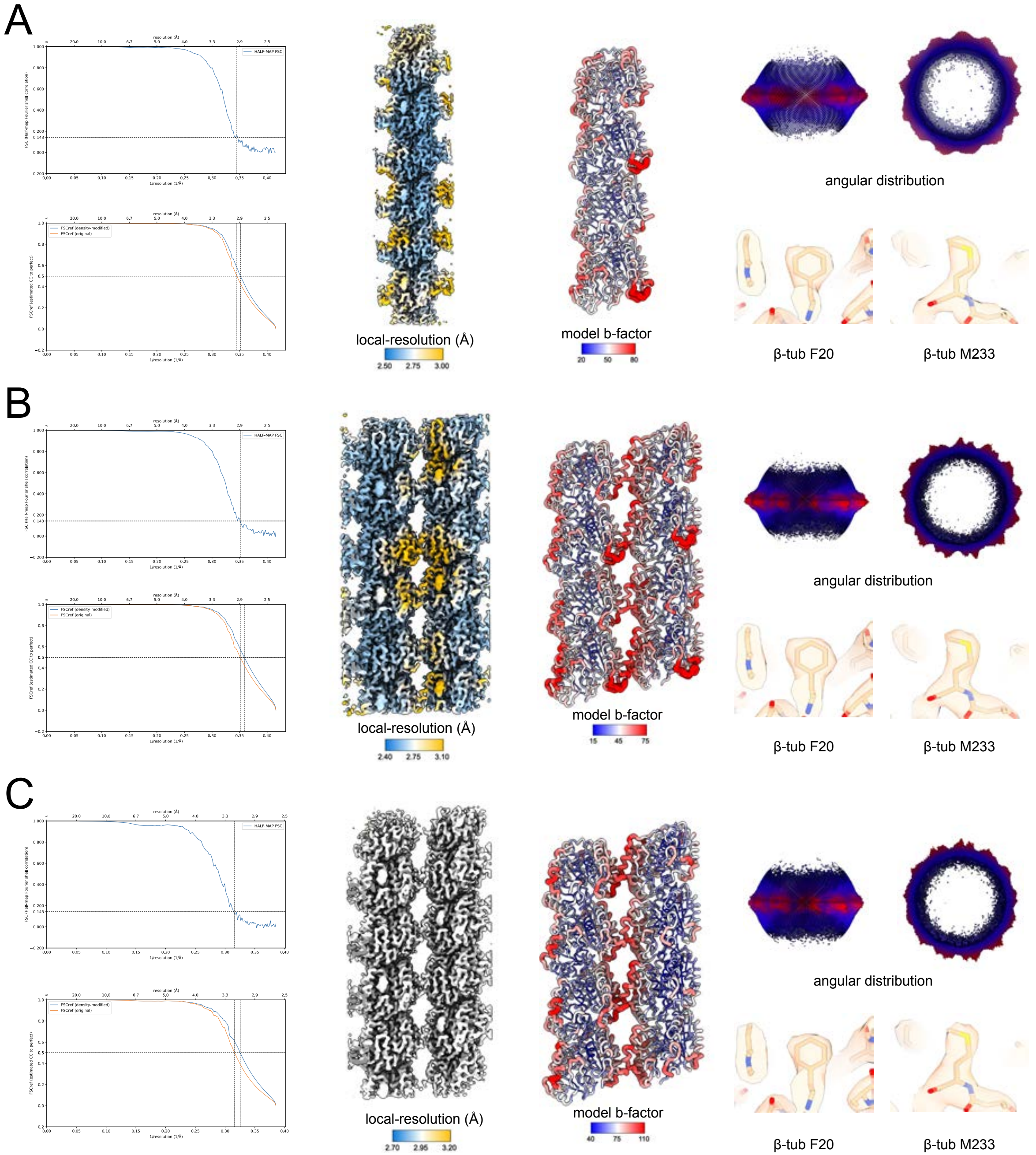

Extended Data Figure 8 - TaccaAJ-GDP 13-protofilament structure validation.

	Tacca-GDP MT		
**Data collection and processing**	1-pf	2-pf (homotypic)	2-pf (heterotypic)
**PDB ID**	XXXX	XXXX	XXXX
**EMDB ID**	XXXXX		
Microscope	Glacios		
Voltage (kV)	200		
Detector	Falcon 4		
Electron exposure (e^-/Å^2)	52		
Frame number	55		
Calibrated pixel size (Å)	0.88838		
Defocus range (μm)	0.5-2.0		
Symmetry imposed	C1		
Movies	9,602		
Initial particle images (before symmetry-expansion)	1,209,568		
Final particle images	1,558,512	1,148,862	397,803
Map resolution (Å)	2.99	2.93	3.26
FSC threshold	0.143	0.143	0.143
Map sharpening factor (Å^2)	-90.2	-86.1	-90.4
Density-modified map resolution (Å)	2.84	2.79	3.07
Density-modified FSC threshold	0.5	0.5	0.5
Helical symmetry (13-pf MT map)			
Single subunit rise (Å)	9.31		
Helical twist (°)	-27.7		
Dimer rise (Å)	80.54		
Axial twist (°)	-0.11		
**Refinement**			
Initial models (AlphaFold ID)			
α-tubulin	PDB 35SN		
β-tubulin	PDB 35SN		
Model resolution (Å)	2.51	2.47	2.68
FSC threshold	0.5	0.5	0.5
Model composition			
Non-hydrogen atoms	13,774	27,548	27,280
Protein residues	1,730	3,460	3,420
Ligands	8	16	12
B factors (Å^2)			
Proteins	47.33	45.28	69.76
Ligands	49.68	43.91	69.52
R.M.S. deviations			
Bond lengths (Å) (# > 4)	0.003 (0)	0.003 (0)	0.004 (0)
Bond angles (°) (# > 4)	0.520 (2)	0.519 (4)	0.569 (7)
Validation			
MolProbity score	1.81	1.80	2.09
Clashscore	7.69	7.91	9.59
Poor rotamers (%)	2.65	2.41	4.19
Ramachandran plot			
Favored (%)	97.68	97.65	97.41
Allowed (%)	2.32	2.35	2.59
Disallowed (%)	0.00	0.00	0.00
Rama-Z score	0.76	1.01	0.88

Table 1 - Cryo-EM data collection, processing, and refinement statistics.
